# Integrating social nutrition principles into the treatment of steatotic liver disease

**DOI:** 10.1038/s43856-023-00398-3

**Published:** 2023-11-10

**Authors:** Dana Ivancovsky-Wajcman, Paul N. Brennan, Christopher J. Kopka, Shira Zelber-Sagi, Zobair M. Younossi, Alina M. Allen, Karen R. Flórez, Jeffrey V. Lazarus

**Affiliations:** 1 Barcelona Institute for Global Health (ISGlobal), Hospital Clínic, University of Barcelona, Barcelona, Spain; 2 Medical Research Institute, Ninewells Hospital, University of Dundee, Dundee, UK; 3 The Global NASH Council, Washington, DC USA; 4 Independent researcher, Ponte de Lima, Portugal; 5 School of Public Health, Faculty of Social Welfare and Health Sciences, University of Haifa, Haifa, Israel; 6 Obesity Research Program, Inova Health System, Falls Church, VA USA; 7 Division of Gastroenterology and Hepatology, Mayo Clinic, Rochester, MN USA; 8 CUNY Graduate School of Public Health and Health Policy (CUNY SPH), New York, NY USA

**Keywords:** Public health, Non-alcoholic fatty liver disease, Non-alcoholic steatohepatitis, Health services, Nutrition therapy

## Abstract

Ivancovsky-Wajcman et al. outline the need for a holistic preventive hepatology approach, involving social nutrition and social prescribing, to address the public health threat of metabolic dysfunction-associated steatotic liver disease (MASLD). They argue that this will facilitate individuals’ engagement in behavioural modifications to treat MASLD.

## Teaser

Current treatment of metabolic dysfunction-associated steatotic liver disease focuses on adjusting patients’ lifestyles, including promoting weight loss and physical activity. Here, we suggest adopting a holistic preventive hepatology approach encompassing social nutrition, social prescribing and broader societal changes to facilitate individuals’ engagement with behavioural modifications.

Formerly known as non-alcoholic fatty liver disease (NAFLD), metabolic dysfunction-associated steatotic liver disease (MASLD) is characterised by fat accumulation in the liver (hepatic steatosis) in the absence of excess alcohol intake, use of steatogenic drugs (medications that promote the accumulation of excess fat in liver cells) or competing hepatic causes, such as viral hepatitis. MASLD can progress to its more aggressive phenotype, metabolic dysfunction-associated steatohepatitis (MASH, formerly known as non-alcoholic steatohepatitis [NASH]), which may predispose some individuals to develop progressive liver fibrosis. This can then advance to cirrhosis and/or hepatocellular carcinoma (HCC) and possibly require liver transplantation. During 2010–2019, MASH was the fastest growing cause of HCC^1^. MASLD is also related to declining cognitive and mental health, particularly depression and anxiety^2^, as well as significant impairment of health-related quality of life^3^.

There are ongoing efforts to develop targeted and effective pharmacological treatments for MASH. However, there are currently no licensed drugs, despite significant advances in the understanding of MASLD and MASH pathogenesis and substantial efforts in the clinical trial space^4^. Given this absence of licensed pharmacological therapy, lifestyle interventions, including those that promote weight loss through lifestyle behaviour change, remain as the first line of treatment^5^ and should be prescribed to all patients, in conjunction with medications targeting commonly associated comorbidities, such as type 2 diabetes (T2D), obesity and cardiovascular disease. If lifestyle adjustments or pharmacotherapy do not lead to the required weight loss, metabolic surgery may be recommended. Nonetheless, this approach has inherent associated risks and is unlikely to be available or suitable for most patients.

Clinical nutrition research provides compelling evidence that specific dietary factors can disturb metabolic homoeostasis either directly, by affecting blood glucose levels, insulin resistance, hepatic steatosis and inflammation levels and the gut microbiome or indirectly, through excessive caloric consumption leading to weight gain. Suboptimal nutrition thus contributes to rising T2D incidence rates, with an estimated 14 million new cases arising between 1990 and 2018 globally^6^, and increases the risk of developing MASLD by 56%, according to a 2021 meta-analysis^7^. A healthy diet is therefore a pivotal and modifiable factor in MASLD development.

Current nutrition approaches for MASLD and MASH focus on achieving a 7–10% weight loss by adopting a healthy diet tailored to patients’ preferences and metabolic comorbidities, if present. Dietary treatment involves reducing the intake of refined grains, fructose, processed meat, saturated fatty acids and alcohol and increasing the consumption of foods typical of the Mediterranean diet, including vegetables, whole grains, omega-3 fatty acids and olive oil. In many cases, caloric intake reduction is also warranted^5^. A healthy diet should be coupled with an adequate exercise programme that reduces sedentary time^8^. However, achieving sustained lifestyle adjustments and weight loss remains a complex challenge for most patients. Barriers are commonly associated with commercial and social determinants that influence an individual’s well-being and health outcomes, such as limited access to healthy lifestyle resources in one’s neighbourhood, including affordable nutritious food, and the cost and time constraints of making sustained lifestyle adjustments.

Commercial and social determinants of health thus impact the prevalence of not just MASLD and MASH but also of other non-communicable diseases (NCDs), such as hypertension, obesity and T2D. While acknowledging that it is important to develop pharmacological agents for MASH and MASLD, just as there are such treatments for other NCDs, we also call attention to the need for increased government, industry and community action in promoting the accessibility of healthy lifestyles. In practice, this can be done in several ways, such as by improving access to affordable nutritious foods via subsidies, discouraging consumption of sugar-sweetened beverages through taxation, reformulating food products to reduce the amount of harmful ingredients, encouraging physical activity by ensuring adequate city infrastructure and promoting employee well-being by, for example, prioritising mental health. These are all necessary components to reduce the incidence of MASLD and NCDs in general and to strengthen MASLD and MASH policies, strategies and guidelines for treatment^9^, in order to end this public health threat.

Health systems should also be equipped to provide behavioural education and tools to improve individuals’ physical, mental and social well-being. However, healthcare professionals in clinical settings often have limited capacity to effectively address the underlying social problems that contribute to both the onset and persistence of poor physical and mental health^10^. The concept of social prescribing, which does not have a universally agreed-upon definition or intervention scheme, tries to fill this gap by connecting individuals with appropriate non-medical resources relevant to their needs to promote overall well-being. It encompasses a range of interventions to promote lifestyle changes relating to factors like smoking, diet and physical activity, as required. Such interventions may include a local jogging group, a list of healthy food substitutes, community gardening, a nutrition education group session, a healthy cooking club or support groups for smoking and/or alcohol cessation or stress management^10^.

To effectively leverage and implement social prescribing interventions, it is crucial to first understand the social dimension of nutrition. Nutrition exists at the interface between medical and social sciences. Social nutrition explores how social factors, including culture, faith and ideology, community, family and support networks influence what, when, how and why individuals eat. This, in turn, impacts health outcomes, including the likelihood of developing NCDs. Social nutrition also considers the nutritional consequences of social processes of globalisation, urbanisation, impoverishment, pandemics, nutrition education and policy^11^. Social determinants of health, including socioeconomic status, migration, dietary culture, place of residence, and gender and commercial factors may also be influential. Taken together, the origins of malnutrition and obesity lie not only in personal food choices and biological mechanisms but in multilayer social environments that may not promote health, especially among vulnerable and minoritised populations. To implement a health-promoting policy that balances biological perspectives and social sciences, policymakers must recognise the importance of social factors in determining the nutritional health and well-being of individuals, households, communities and populations, across the lifespan^11^.

Implementing a holistic approach, focusing on both prevention and treatment of MASLD and MASH, and their associated comorbidities, requires the involvement of multiple stakeholders from diverse medical specialisations beyond hepatology, such as endocrinology, cardiology, nutrition, obesity and mental health. Efforts to prevent MASLD should commence at the earliest stages, requiring the involvement of maternal medicine and paediatrics. It also warrants the participation of public health professionals, policymakers, governmental bodies, community leaders and industry in implementing change. In addition, it is necessary to involve the education system and implement realistic and sustainable healthy nutrition policies in schools in order to prevent childhood obesity and MASLD. Hepatology associations can learn from cardiology associations^12^ in, for instance, advocating for healthier food systems, thus ensuring that primary prevention is a central tenet of preventive hepatology^13^.

Recent evidence highlights the challenges of managing complex co-morbid diseases, such as T2D and MASLD. In a 2023 randomised trial, despite implementation of conventional best-practice approaches, including optimised pharmacological therapy and lifestyle interventions, patients achieved minor weight loss and poorer liver-related improvements than those undergoing metabolic surgery^14^. Given the high prevalence of T2D and MASLD, the cost of surgical interventions and the limited capacity of healthcare systems, only a small proportion of patients receive adequate care. Integrating social prescribing and nutrition interventions as part of the prevention and treatment of MASLD and other NCDs may reduce the burden on health services while improving patients’ physical and mental health.

**Fig. 1:**
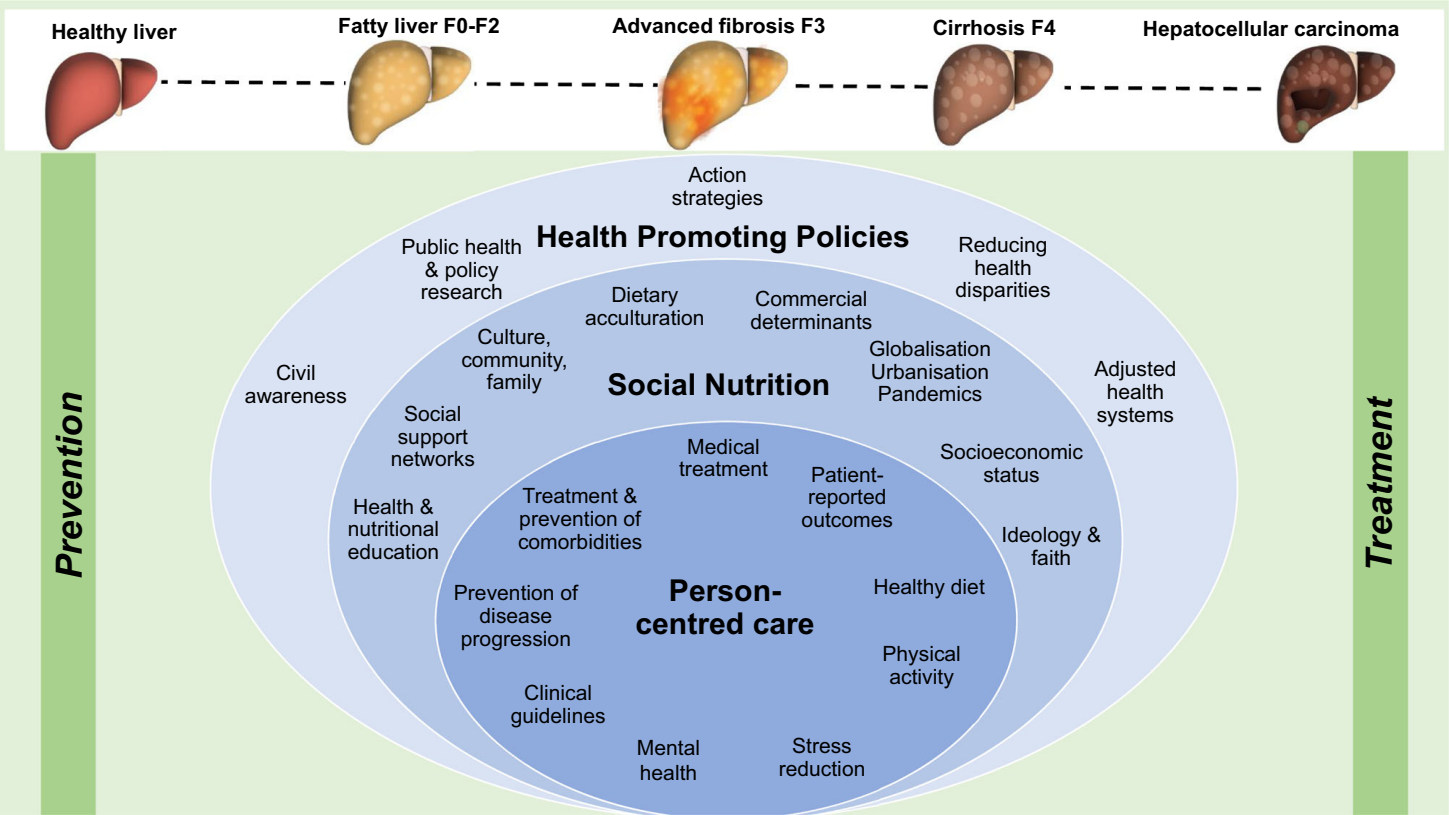
Achieving favourable steatotic liver disease outcomes via social nutrition-informed preventive hepatology. The implementation of person-centred care, social nutrition and health-promoting policies is central in preventing and treating steatotic liver disease. Steatotic liver disease exists as a continuum from simple steatosis to cirrhosis and fibrosis progression (F-stage) is associated with poorer outcomes. Fibrosis progression is not unidirectional and prior to cirrhosis it is reversible through lifestyle changes, glycaemic control and weight loss. Hepatocellular carcinoma may develop at any fibrosis stage, but can be prevented with a healthy lifestyle.

To achieve favourable health outcomes, health systems must adopt holistic, personalised and person-centred care, combined with ambitious policy and social interventions (Fig. 1). We believe that these strategies complement pharmacological therapies currently in trials and in real-world settings, after regulatory approval, and would be a crucial part of a person-centred approach to tackling MASLD^15^. These synergistic efforts would help to reduce the incidence and prevalence of MASLD and MASH and associated metabolic diseases, as well as reducing their economic and societal impact.
